# Effects of Dispositional Mindfulness and Mindfulness-Based Interventions on the Psychosocial Consequences of Burn Injuries: A Systematic Review

**DOI:** 10.3390/ebj6020025

**Published:** 2025-05-15

**Authors:** Luca Simione

**Affiliations:** 1 Dipartimento di Scienze Umanistiche e Sociali Internazionali, Università degli Studi Internazionali, 00147 Rome, Italy; luca.simione@unint.eu or luca.simione@cnr.it; 2 Istituto di Scienze e Tecnologie della Cognizione, Consiglio Nazionale delle Ricerche, 00196 Rome, Italy

**Keywords:** mindfulness, burn, burn survivors, meditation, yoga, anxiety

## Abstract

Burn injuries lead to significant physical and psychological consequences, including chronic pain, post-traumatic stress, depression, and social isolation. Mindfulness-based interventions (MBIs) have been proposed as a holistic approach to address these challenges in burn rehabilitation. This systematic review evaluates the efficacy of dispositional mindfulness and MBIs, including mindfulness meditation, yoga, and self-compassion training, in managing pain, emotional distress, and psychosocial adaptation in burn survivors. A comprehensive literature search was conducted through MEDLINE and Web of Science, covering studies up to February 2025, with additional papers retrieved from Google Scholar and Semantic Scholar. Studies were included if they reported quantitative data on the effects of MBIs in burn patients and/or their families, excluding opinion pieces, editorials, reviews, and qualitative studies. After screening 91 studies retrieved from the databases and adding a compelling paper retrieved from the other sources explored, 12 studies were included in the final pool, categorized into cross-sectional studies (*n* = 6), and intervention studies (*n* = 6). The extracted data included publication year, research design, sample characteristics, intervention details, main findings, and data for quality assessment. The synthesis of the results suggests that mindfulness is associated with reduced psychological symptoms, improved emotional regulation, and enhanced self-compassion, leading to better coping strategies and social reintegration. However, the long-term efficacy of MBIs remains inconclusive, and further research is needed to differentiate mindfulness-specific effects from those of general physical exercise. Evidence also suggests that mindfulness interventions may reduce anxiety and secondary trauma in children with burns and their caregivers. This review highlights the potential of MBIs as adjuncts to conventional burn rehabilitation programs, but further high-quality trials are needed to establish their sustained efficacy and to understand the specific benefits of mindfulness.

## 1. Introduction

Burn injuries represent a complex psychophysical condition, intertwining severe physical damage with profound psychological impacts. The aftermath of a burn is not confined to the immediate physical pain and healing process but extends into chronic pain, mental health challenges like post-traumatic stress disorder (PTSD) and anxiety, and significant social and familiar disruptions [1]. Understanding burns as a condition that affects both body and mind underscores the need for comprehensive treatment approaches that address the full spectrum of burn survivors’ experiences. In the last years, mindfulness has emerged as a compelling and holistic treatment for the whole spectrum of psychophysical symptoms experienced by burn survivors and their relatives [2]. In light of this, a systematic review was conducted to put together all the existing evidence in favor of such applications and investigate the strengths and weaknesses of this literature.

One of the most common consequences of burn wounds is chronic pain, which persists long after the initial wounds have healed [3]. This ongoing pain can be attributed to several factors, including nerve damage, the development of scar tissue, and the prolonged inflammatory response associated with severe burns. For many survivors, pain not only is a physical burden but also exacerbates emotional distress and limits their ability to engage in daily activities [4]. Studies have shown that the prevalence of chronic pain among burn survivors is remarkably high, affecting their quality of life and often requiring long-term management strategies. The complexity of burn-induced chronic pain highlights the need for multidisciplinary approaches that address both the physiological and the psychological dimensions of pain in this population.

PTSD is another common psychological consequence among burn survivors, with symptoms that can manifest long after the physical wounds have healed [5]. PTSD in burn survivors is often characterized by intrusive memories of the traumatic event, flashbacks, hypervigilance, and a pervasive sense of anxiety. The incidence of PTSD among burn survivors is influenced by the severity of the injury, the individual’s pre-existing mental health status, and the support systems available during recovery. The psychological toll of a burn injury can be just as debilitating as the physical damage, necessitating comprehensive mental health care as part of the recovery process [6,7]. The trauma associated with burn injuries frequently leads also to depression and anxiety, conditions that can significantly impair a survivor’s ability to cope with their new reality [8,9,10]. Depression in burn survivors often stems from the drastic changes in their appearance and function, the loss of independence, and the chronic pain that accompanies recovery. Anxiety, on the other hand, may arise from fear of re-injury, social stigmatization, or the stress of ongoing medical treatments. The two conditions are interlinked, creating a vicious cycle that can hinder recovery and diminish the overall quality of life. Addressing these mental health challenges is critical to ensuring holistic recovery for burn survivors [11].

Another path of suffering for burn survivors could be represented by the significant social challenges they should cope with, with survivors experiencing varying degrees of social isolation [12]. This isolation can be due to several factors, including visible disfigurement, physical limitations, and the psychological impact of the injury. Survivors may withdraw from social interactions, fearing judgment or rejection due to their altered appearance. This withdrawal can lead to a sense of loneliness and exacerbate the feelings of depression and anxiety. Moreover, the stigmatization and societal perceptions of burn injuries can further alienate survivors, making social reintegration a complex and challenging process [13,14].

Such psycho-social impact of a burn injury extends beyond the survivor, profoundly affecting family members and caregivers. Families often bear the emotional and financial burden of care while navigating the complexities of long-term recovery and rehabilitation [15]. Caregivers, in particular, may experience significant stress and burnout, as they provide continuous support while grappling with the emotional toll of their loved one’s injury [16]. The dynamics within a family can shift dramatically, with roles and responsibilities changing to accommodate the needs of the burn survivor. This can lead to strained relationships, reduced social interactions, and an overall decline in family well-being [17].

Considering all the psychological and social consequences of burn injuries, there is a compelling need for an integrated psychological treatment as a core component of burn care [18]. Psychological interventions should address not only the mental health conditions commonly associated with burns, but also the broader issues of self-esteem, social reintegration, and family dynamics. Effective psychological treatment can significantly enhance the overall recovery process, improving both mental and physical outcomes for burn survivors. This holistic approach is essential for helping survivors rebuild their lives and regain a sense of normalcy after their injuries [19].

In recent years, mindfulness has emerged as a promising psychological intervention for managing the complex array of symptoms experienced by burn survivors [2]. Mindfulness-based interventions (MBIs) focus on cultivating present-centered awareness and acceptance of experiences, which can be particularly beneficial in coping with chronic pain, emotional distress, and the psychological aftermath of trauma [20,21,22]. As such, mindfulness represents a valuable tool in the psychological treatment of burn survivors, offering a non-invasive and holistic approach to recovery [23]. Mindfulness is a psychological construct that encompasses two primary components: awareness and acceptance. According to the model proposed by Bishop et al. [24], mindfulness involves paying attention to the present moment in a non-judgmental manner. This heightened awareness allows individuals to observe their thoughts, emotions, and sensations without becoming overwhelmed by them. Acceptance, the second component, encourages an open and non-reactive attitude towards one’s experiences, fostering resilience in the face of adversity. These elements of mindfulness are integral to its therapeutic potential, particularly in helping individuals manage stress, pain, and emotional difficulties [22,25]. While Bishop et al. [24] laid the groundwork for mindfulness by defining it as awareness and acceptance, more recent theories have refined and expanded this framework to better understand its role in emotional regulation and recovery.

A key development is the Monitoring and Acceptance Theory (MAT) proposed by Lindsay and Creswell [26,27], which emphasizes the dynamic interplay between monitoring and acceptance in mindfulness practice. Monitoring involves observing internal experiences, while acceptance refers to embracing these experiences as they are. According to this theory, the combination of these two processes allows individuals to regulate their emotional responses more effectively, reducing psychological distress [28]. This process of acceptance and emotional regulation also ties into the concept of self-transcendence, as described by Garland and colleagues [29,30]. Through mindfulness, individuals are able to move beyond self-centered concerns, particularly those tied to suffering or trauma, and experience a greater sense of meaning and purpose. By transcending the ego and finding meaning in their experience, individuals can rebuild a sense of identity and purpose, even in the face of significant hardship. Complementing this idea is Neff’s self-compassion model [31], which introduces the importance of treating oneself with kindness, especially during times of suffering. Self-compassion involves recognizing that suffering is part of the shared human experience and offering oneself care and understanding rather than self-criticism. For burn survivors, practicing self-compassion can mitigate the feelings of shame and self-blame often associated with trauma, promoting emotional healing and acceptance of their altered bodies and experiences [32]. By integrating these theories, we can better understand how mindfulness can support burn survivors.

The mapping of these distinct yet complementary models to different types of mindfulness practices highlights the diverse approaches available for addressing the varied psychological needs of burn survivors. For example, a particular class of practices is represented by yoga-based mindfulness practices, such as Hatha yoga or Pranayama, which integrate physical movement, breath control, and meditation to foster mindfulness and emotional regulation. These practices have been proved as effective in reducing a variety of psychological symptoms, such as depression and anxiety [33,34]. Moreover, yoga practice is also embedded in the most widely adopted MBIs, i.e., mindfulness-based stress reduction [33,35] and mindfulness-based cognitive therapy [36].

Regardless of the theoretical model used, mindfulness can be understood both as a trait and as a set of practices. Dispositional mindfulness is defined as a person’s natural tendency to be aware of and attentive to the present moment in a non-judgmental or accepting way. Meditative practices such as mindfulness meditation, body scan, and breathing exercises aim to enhance one’s capacity for mindfulness [29,37]. Such an increase in mindfulness has been related to improved emotional regulation, stress reduction, and overall psychological well-being [38,39]. The adaptability of mindfulness practices makes them suitable for a wide range of therapeutic contexts, including the rehabilitation of burn survivors.

Mindfulness has been shown to have a significant impact on pain management, particularly in chronic pain conditions. By fostering a non-reactive awareness of pain sensations, mindfulness allows individuals to reduce the emotional and cognitive distress associated with pain. This can lead to a reduction in the perceived intensity of pain and an increase in pain tolerance [40,41]. Mindfulness is also credited for a general effect on mental health, which has been well-documented [22]. MBIs have been shown to reduce psychological symptoms by promoting greater emotional regulation, reducing rumination, and enhancing overall psychological resilience. For burn survivors, mindfulness could then offer a way to cope with the intense emotional aftermath of their injuries, helping to mitigate the effects of trauma and facilitate a more positive outlook on recovery. Moreover, mindfulness could also positively influence the social lives of burn survivors by promoting greater self-acceptance and reducing the stigma associated with their injuries [42,43,44]. Through mindfulness practices, survivors could develop a more compassionate relationship with themselves, which can translate into more confident social interactions and reduced social isolation [31].

To summarize, mindfulness could help people suffering from burn injuries and their families, aiding on both sides of pain management, psychological sequelae, and social difficulties. While the application of mindfulness interventions to patients with burn injuries is in its infancy, a systematic review of the studies involving mindfulness and mindfulness practices in this kind of patients was conducted. The aim of this systematic review was to assess which of the hypothesized paths of mindfulness effects on the psychosocial well-being of patients with burn injuries have been explored in the literature and if such positive effects have been confirmed or not. From this analysis, a general picture of the actual literature could be drawn to then derive some practical advice to guide the further development of specific mindfulness-based interventions for burned patients and their caregivers, as well as to conduct future research in the field.

## 2. Materials and Methods

**Figure 1:**
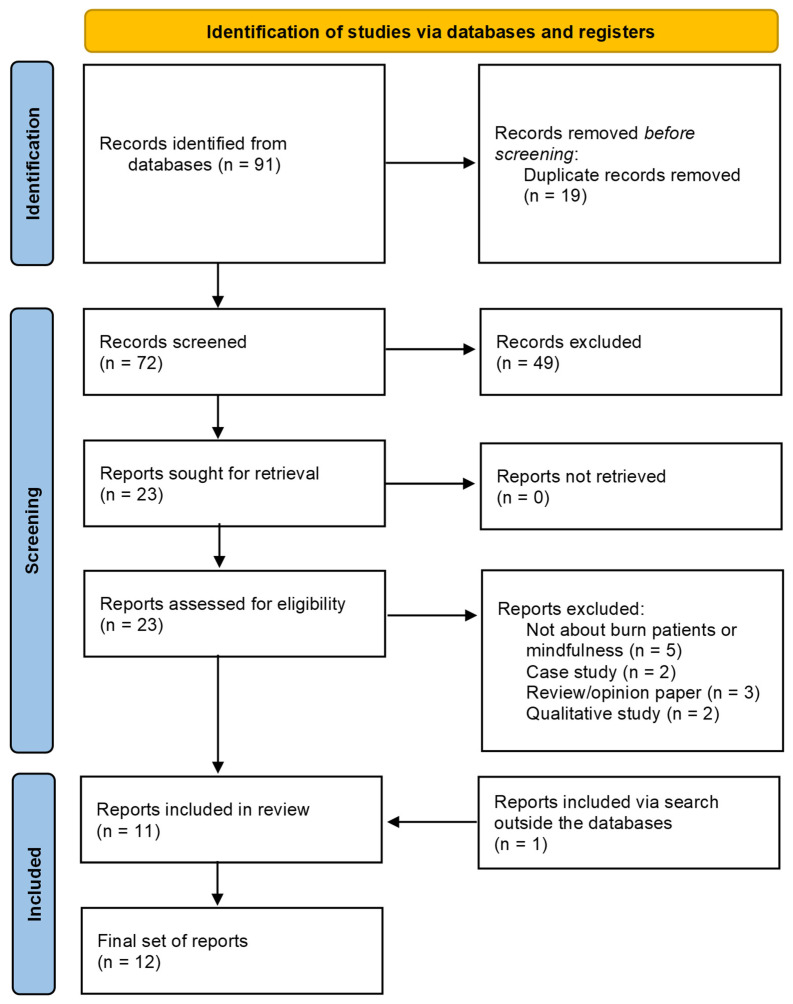
PRISMA flow diagram illustrating the selection process of studies for inclusion in this systematic review. The chart outlines the stages of identification, screening, eligibility, and inclusion, detailing the number of records identified through database searching, the number of records after duplicates were removed, the number of records screened, full-text articles assessed for eligibility, and the final number of studies included in the qualitative synthesis.

This systematic review was conducted according to the Preferred Reporting Items for Systematic Reviews and Meta-Analyses (PRISMA) guidelines, ensuring a transparent and replicable approach [45]. A protocol was developed for this systematic review, which was registered via OSF at https://doi.org/10.17605/OSF.IO/DGP4E (accessed on 14 March 2025). The stages for the identification, screening, and inclusion of the studies of interest are shown in Figure 1. There were no significant deviations from the protocol during the review process.

### 2.1. Eligibility Criteria

This systematic review focused on selecting manuscripts that reported correlational or intervention studies on mindfulness applied to patients with burn injuries and their families. Interventions were considered mindfulness-based if they included specific mindfulness instructions or practices, rather than solely focusing on physical aspects, as for some yoga interventions. Case studies, opinion articles, editorials, reviews, and, in general, papers not reporting new data and results were excluded from the review. To ensure methodological rigor, only studies that explicitly utilized quantitative methodologies were included. Papers considered for this review should be written in English, empirical in nature, and had undergone peer review. No restrictions were placed on participant demographics, ensuring the inclusion of studies regardless of factors such as age, sex, socioeconomic status, or year of publication. This approach was designed to facilitate a broad and comprehensive analysis of the available empirical evidence.

### 2.2. Search Strategy

A literature search was conducted through two databases: MEDLINE and Web of Science. The search was limited to papers published up to February 2025 (date of the database search). A unified search string employing the Boolean operators ‘OR’ and ‘AND’ was defined as follows: (‘burn’ OR ‘burn injury’ OR ‘burn wounds’) AND (‘mindfulness’ OR ‘yoga’ OR ‘compassion’ OR ‘MBSR’ OR ‘MBCT’) NOT (‘burnout’ OR ‘burn out’). The terms ‘yoga’ and ‘compassion’ were included to cover the broader field of mindfulness interventions. In fact, yoga practices often incorporate mindfulness elements, such as focused attention and body awareness. Also ‘compassion’ was included because self-compassion is increasingly recognized as a core component of mindfulness-based interventions and is relevant to the psychological well-being of burn survivors. MBSR and MBCT instead refer to the most common mindfulness-based interventions. This search string was applied to all fields throughout the databases investigated.

The retrieved items were further integrated through a compelling search conducted on both Google Scholars and Semantic Scholar, which was carried out to identify papers missing from the main scientific databases or grey literature. Grey literature was included in the search to identify any potentially relevant studies that may not have been published in peer-reviewed journals, such as conference proceedings or dissertations. This was done to provide a more comprehensive overview of the available evidence, particularly in this emerging field of research.

### 2.3. Study Selection

The selection process was conducted in accordance with the PRISMA guidelines [45]. The broad search string, applied over all fields, produced a total of 91 papers. Nineteen duplicates were identified and removed, and then the inclusion and exclusion criteria were applied. After inspection of the title and abstract, 49 further papers were removed because irrelevant. The remaining 23 papers were retrieved in full text to be analyzed. From this pool, 12 other studies were removed, in particular, 2 case studies, 2 opinion letters, 1 review paper, 5 papers not including burn themes or not in relation to mindfulness, and 2 qualitative studies. Another relevant paper was added to this pool, which was retrieved by exploring the other sources (Google Scholar, Semantic Scholar). At the end, a total of 12 relevant studies were included in the review.

### 2.4. Data Extraction

For each study, the following information was extracted: year of publication, author(s), research design, sample characteristics and size, intervention/instruments, main findings/outcomes, and data for quality evaluation (see next section).

### 2.5. Risk of Bias Assessment

Due to the wide variety of studies included in this systematic review, a formal risk of bias assessment was conducted using the NIH study quality assessment tool. To accommodate both correlational and intervention studies, a set of items from the tool were adapted as follows in order to evaluate eleven potential sources of bias: research question (was the research question or the objective in this paper clearly stated?); study population (was the study population clearly specified and defined?); participation and drop-out (was the participation rate of eligible persons at least 50%, and was the drop-out rate reasonable for intervention studies?); participant selection (were all subjects selected or recruited from the same or similar populations? Were the inclusion and exclusion criteria for participation prespecified and applied uniformly to all participants?); sample size (was a sample size justification, power description, or variance and effect estimate provided?); timeframe of the study (was the timeframe sufficient to reasonably expect an effect, if one existed?); reliable assessment and statistics (were the independent variables clearly defined, valid, reliable, and implemented consistently across all participants? Was the data analysis properly described and conducted?); presence of multiple assessments/follow-up (were the outcomes assessed more than once over time and/or was any follow-up conducted for intervention studies?); assessment of outcomes (were the outcome measures clearly defined, valid, reliable, and implemented consistently across all participants?); blindness (were the outcome assessors blinded to the participants’ exposure status?); and control for confounders (were key potential confounding variables measured and statistically adjusted for their impact on the outcomes?).

For each considered dimension, a score of 0 was assigned if the paper did not adequately address that dimension, a score of 1 if relevant information was reported, and the ‘N’ mark if the evaluation of the dimension was not feasible based on the information available in the paper. The overall quality of each study was then evaluated based on its total score as poor (less than 6), fair (between 6 and 8), or good (greater than 8). Irrespective of their quality, all studies were retained in the pool considered for this systematic review. Table 1 reports a summary of the bias evaluation for each study and its overall assessment.

### 2.6. Data Synthesis

Due to the heterogeneity of the included studies in terms of study design, intervention types, and outcome measures, a meta-analysis was not feasible. Instead, a narrative synthesis approach was employed. This involved systematically organizing the findings from each study, identifying common themes and patterns across the studies, and summarizing the evidence. The studies were grouped based on the type of intervention (e.g., mindfulness meditation, yoga) and the primary outcome (e.g., pain, anxiety) to facilitate their comparison. The initial phase of the review involved screening titles and abstracts using the predefined search terms to ensure alignment with the eligibility criteria. Subsequently, a more in-depth analysis of the selected papers was conducted. The studies included in the review were categorized mainly by research design, i.e., correlational or interventions. Through this process, two final groups of papers were obtained, divided as follows: cross-sectional studies (*n* = 6), and intervention studies (*n* = 6). The correlational studies were further divided based on the mindfulness construct considered, i.e., mindful awareness (*n* = 4) and self-compassion (*n* = 2). The intervention studies were further classified based on the main type of practice included in the intervention, i.e., mindfulness (*n* = 3) or yoga (*n* = 3). In this review, yoga practices refer to interventions that mainly emphasize physical or movement components, such as asana exercises, without significant mindfulness elements. The information retrieved from the set of papers included in the review is reported in Table 2.

## 3. Results

### 3.1. Cross-Sectional Studies

This group included six papers describing the results of correlational studies mainly based on questionnaires and self-report measures, assessing trait-like mindfulness facets. In particular, they focused on mental presence assessed with the MAAS [46], self-compassion assessed with the Self-Compassion Scale [31], and the acceptance stance of mindfulness assessed with either the Acceptance and Action Questionnaire II (AAQ-II, [47]) or the Five-Facets Mindfulness Questionnaire (FFMQ, [48]). The studies considered a number of different outcomes such as psychological distress, appearance anxiety, and quality of life. The majority of the studies included were conducted on adult burn patients, while only one focused on children and their parents [49]. Of note, two papers reported results obtained from the same sample of burn survivor [50,51].

Overall, this set of studies showed a positive effect of mindfulness skills on the assessed outcomes. Mindful attention and awareness correlated negatively with psychological distress [51,52] and positively with burn-specific QOL [50], while the acceptance and self-compassion facets were more related to reduced appearance anxiety [53,54] and depression [49]. The only study assessing the effect of dispositional self-compassion on the mental health of caregivers and parents of children with burn injuries [49] found a positive effect on both depressive and post-traumatic symptoms, but not on anxiety. Based on this body of evidence, the dispositional mindfulness facets seem to have a positive effect on core aspects of the distress associated with burn injuries, increasing cognitive and emotional strategies to cope with such an impactful event.

### 3.2. Mindfulness Interventions

This group included three papers with intervention protocols applied to both adult burn patients [55,56] and parents of children with burn injuries [57]. Only one study exploited a widely used mindfulness-based protocol [56], i.e., mindfulness-based stress reduction [58,59]. Another study, instead, combined psychoeducational aspects with mindfulness and visualization exercises [57], whereas the last one combined mindfulness-based techniques such as breathing meditation and body scan with a self-care nursing protocol [55]. Moreover, one study used an RCT design [57] with a control group, whereas the other two did not. The major limitations of this set of studies are their quasi-experimental design, with lack of a control group and randomization [55,56], and the use of inactive control groups such as waiting list groups [57]. They also included a small-to-medium sample size, ranging from 17 [56] to 62 [57] patients, with limitations in the generalizability of their results. They assessed the effect of such interventions on a number of different outcomes including post-traumatic stress symptoms, self-esteem, QOL, mindfulness, and self-compassion.

Overall, they found a positive effect of mindfulness exercises on psychophysical factors such as anxiety, pain, and stress, and a concomitant increase in self-esteem and mindfulness skills such as self-compassion. While two of such interventions reported an effect on outcomes also at a three-month follow-up [56,57], they failed in consistently finding such results at longer follow-up assessments [57].

### 3.3. Yoga Interventions

This group also included three papers with intervention protocols applied to both adults [60,61] and children with burn injuries [62]. One study involved an intensive 4-day yoga intervention without a control group [62], while the other two studies included a 4-week yoga practice in the form of yoga Nidra [60] or Pranayama breathing exercise [61]. Moreover, these two latter studies adopted an RCT design with a control group. Differently from the typical mindfulness-based interventions, those yoga-based interventions included guided practice multiple times per week. The range of outcomes assessed within this group of papers included both somatic and psychological factors. The samples included were of moderate size, comprising from 30 [61] to 110 participants [60,61].

Overall, yoga interventions showed promising results in burn survivors. In fact, based on the reviewed literature included in this analysis, yogic practices resulted in improved psychological conditions in both children [62] and adult patients [60,61]. These practices also had a positive impact on certain health outcomes, such as pain intensity and bodily functions [61]. The only study with a follow-up assessment also found that the positive effect persisted after a three-month interval [61].

## 4. Discussion

Mindfulness-based interventions (MBIs) have gained increasing attention in the rehabilitation of burn survivors due to their holistic approach to addressing both psychological and physiological challenges. The findings of this systematic review indicate that mindfulness, defined as present-centered awareness and acceptance, has significant potential to mitigate distress in burn patients. Despite these promising findings, research on MBIs for burn patients is still in an early phase, with a limited number of randomized controlled trials specifically targeting this population and related individuals, such as family members or caregivers. Many existing studies rely on self-report measures, small sample sizes, or quasi-experimental designs, which limits the generalizability of the results [51,54]. Additionally, while mindfulness appears to enhance coping mechanisms, there is still a lack of robust evidence demonstrating that it is superior to other psychological interventions, such as cognitive–behavioral therapy or exposure-based treatments, for burn survivors [63]. Therefore, although MBIs show promise as complementary interventions in burn rehabilitation, the current findings should be considered preliminary. Further research, including well-designed randomized controlled trials with larger samples, is needed to validate these results and determine the optimal application of mindfulness interventions in burn care. Then, additional studies are necessary to establish their efficacy, particularly in comparison with other established psychological treatments.

The correlational studies reviewed offer valuable insights into the potential associations between dispositional mindfulness and specific psychosocial outcomes in burn survivors. For example, research has indicated an inverse relationship between dispositional mindfulness and appearance-related anxiety [53]. This suggests that higher levels of trait mindfulness may be associated with reduced distress related to altered body image following burn injuries. This protective effect of mindfulness, particularly when conceptualized in terms of acceptance and self-compassion, is further supported by a prospective study [54]. Similarly, other studies have explored the relationship between dispositional mindfulness and general psychological distress [49,50,51,52]. The available evidence consistently points to a beneficial association, indicating that greater mindfulness may foster a more adaptive response to the psychosocial challenges associated with burn injuries. These findings highlight the potential relevance of dispositional mindfulness in influencing key aspects of psychosocial adjustment among burn survivors.

The relationship between specific components of mindfulness and psychosocial outcomes in burn survivors appears to be nuanced and warrants further investigation. The reviewed correlational studies suggest that dispositional mindfulness is negatively associated with appearance-related anxiety in this population [53]. This finding implies that individuals with higher trait mindfulness may be better equipped to cope with body image concerns, which potentially eases the social difficulties commonly experienced following burn injuries [12,13]. In addition, dispositional self-compassion emerged as a relevant factor in managing both psychological [49] and social [54] challenges faced by burn survivors. However, to date, no intervention study has specifically targeted self-compassion practices, such as compassion-focused therapy [64] or self-compassion training [31,32], within this clinical context. Consequently, while these findings are theoretically grounded and encouraging, they should be interpreted with caution, given their exclusive basis in cross-sectional evidence.

In burn survivors, mindfulness-based self-compassion training could help shift the focus from self-judgment to self-acceptance, reducing the psychological burden of appearance-related distress and enhancing emotional well-being [54]. Self-compassion, as conceptualized by Neff [31], involves treating oneself with kindness, recognizing common humanity, and maintaining mindful awareness in the face of suffering. A more recent conceptualization of compassion further encompasses its holistic capability of including as a construct physical, psychological, and social/situational aspects of individuals [65]. Studies suggest that individuals who cultivate self-compassion are better equipped to manage negative thoughts about their appearance and are less likely to engage in self-criticism [32,66]. Similarly, interventions that integrate self-compassion with cognitive–behavioral strategies have been linked to reduction in body dissatisfaction and improvements in self-esteem and reduced appearance-related distress in individuals with visible differences [67]. Furthermore, by reducing self-criticism and appearance anxiety, mindfulness may facilitate social reintegration by helping burn survivors develop greater comfort in social interactions. Social withdrawal is a common coping mechanism for those who fear judgment based on their appearance [68]. Mindfulness-based approaches help individuals focus on present-moment experiences rather than ruminating on perceived negative evaluations from others [44]. Research on social anxiety disorder suggests that mindfulness can help individuals break free from negative self-referential thought patterns, which could similarly benefit burn survivors facing social stigma [69].

The intervention studies reviewed highlight several key benefits of mindfulness for burn survivors. First, mindfulness practices train individuals to observe their thoughts and emotions without over-identifying with them. This, in turn, leads to improved emotional regulation [70,71]. This is particularly relevant for burn survivors, who often struggle with trauma-related anxiety and depressive symptoms [7]. This effect of mindfulness meditation practice has also been shown to influence the brain’s pain-processing pathways, leading to reductions in perceived pain intensity [72]. In burn patients, mindfulness techniques help disrupt the cycle of pain-related catastrophizing and anxiety, providing a non-pharmacological tool for pain relief. Studies incorporating yoga-based interventions, such as those by Nambi et al. [61], suggest that yogic breathing exercises enhance the respiratory function and reduce pain perception in burn survivors. This is further supported by a case study [73] that examined the use of yoga-inspired therapy in burn rehabilitation in a 38-year-old male with deep partial-thickness electrical burns who underwent yoga therapy four times per week. Over 30 sessions, cervical and shoulder mobility improved. Unfortunately, this study did not consider the psychological aspects of the case; therefore, its relevance in this specific review is limited.

A key consideration in evaluating the effectiveness of MBIs is the durability of their effects over time. While several studies demonstrated positive outcomes immediately following MBI programs and short-term follow-up assessments [57,61], the long-term maintenance of these benefits remains inconsistent. Specifically, one study documented a decline in positive outcomes at a one-year follow-up [57], suggesting a possible waning of the treatment effects, whereas other studies found that benefits were maintained at a three-month follow-up [57,61]. This discrepancy may be attributed to factors such as inconsistent mindfulness practice after the intervention, the challenges of preventing symptom relapse in the context of chronic burn-related issues, or the absence of robust strategies to integrate mindfulness into ongoing rehabilitation. To address this, future research should investigate methods to support a sustained mindfulness practice and enhance the durability of the treatment effects. For example, in a case study, Gomez et al. [74] used a virtual reality setting to deliver DBT-based mindfulness training aimed at enhancing awareness. The subject was an adult male with severe skin burns sustained during a fire, who subsequently developed anxiety symptoms and ruminative thoughts related to his social life and appearance. In this VR-based context, which was also effective for the outcomes considered, the participant reported a willingness to continue meditating outside of the programmed sessions. This suggests that online or immersive virtual environments could represent promising strategies for fostering a long-term engagement in mindfulness practice [75,76].

From the reviewed studies, mindfulness has shown promising effects also in alleviating caregiver burden by reducing feelings of overwhelmingness and improving their ability to provide stable support to a child with burn injuries [56,57]. This effect could rely mainly on an increase in self-compassion skills, as strongly suggested by some of the reviewed studies [49,56]. Increasing mindfulness skills seems also to have a direct impact on children with burn injuries. In fact, a 4-day yoga intervention was sufficient to reduce somatic and cognitive anxiety in such patients [62]. While the existing research supports the value of MBIs in reducing caregiver stress and enhancing family dynamics, more studies are needed to determine the most effective formats, frequencies, and durations of the interventions.

Based on the body of evidence reviewed, several clinical and practical recommendations can be proposed. First, brief mindfulness exercises (e.g., 5 min breathing practices) should be integrated into post-discharge care plans to support patients’ ongoing emotional well-being. Even among non-meditators, very brief daily practices appear to be effective in enhancing mindfulness and promoting positive neuroplastic changes [77,78]. Moreover, a brief practice help maintain such practice over time [79]. A regular yoga practice may also be recommended for specific physical challenges, such as motor difficulties, pain management, and affected pulmonary function [73]. This approach can help patients manage bodily symptoms while also fostering acceptance of their new existential condition and potentially reducing social stigma. Second, mindfulness-based interventions should be developed and evaluated specifically to address appearance-related distress in burn survivors, incorporating techniques focused on body image acceptance. Based on the evidence gathered thus far, a dedicated MBI for burn survivors should primarily include practices aimed at increasing acceptance and self-compassion, which have emerged as key components in addressing the psychosocial difficulties commonly experienced by individuals with burn injuries.

### 4.1. Limitations

The findings discussed so far underscore the potential of MBIs as a valuable adjunct to traditional rehabilitation approaches. However, as discussed in the following section, not all expected benefits have been consistently supported by empirical research. While mindfulness has shown promise in addressing psychological distress and pain management, several anticipated benefits have not been strongly supported by empirical evidence. Several intervention studies reported short-term reductions in stress and anxiety following mindfulness-based programs [56,57]. However, the durability of these effects remains unclear. In some cases, the benefits of mindfulness training were not maintained at long-term follow-ups [57], suggesting that ongoing practice may be necessary to sustain psychological improvements. By the way, there is limited but promising evidence that burn survivors would continue mindfulness practice independently after the end of an intervention (as reported in [74]).

Another path not well pursued by the actual literature is the assessment of the benefits of mindfulness distinguished from those of physical movement. In particular, this applies to the reviewed interventions that were mainly based on yoga and make it difficult to disentangle the benefits of mindfulness from those of mere physical movement [61,62]. While mindfulness involves cognitive processes distinct from those of exercise, the overlap between the two in burn rehabilitation settings requires further investigation. In particular, this can be done by applying well-known mindfulness-based interventions such as MBSR or MCBT that include, but are not limited to, yoga exercises.

A last limitation that emerged from the set of studies included in this review is the lack of differentiation between mindfulness skills. Trait mindfulness, in fact, is usually modeled as a multifaceted construct [24,48], and the combination of its different components could alter the pattern of functioning of mindfulness [27,28]. As MBIs are conceptualized to target and increase the presence of specific mindfulness skills [29,37], understanding how the dispositional presence of such skills in individuals could alter the adaptation response to burn injuries could greatly help in developing more compelling interventions.

### 4.2. Future Studies

These limitations in the literature highlight the need for more rigorous research to refine our understanding of mindfulness in burn recovery. Future research should focus on assessing whether MBIs provide lasting psychological and physiological benefits for burn survivors. Follow-up assessments at six months, one year, and beyond would help determine the persistence of mindfulness-related improvements.

While mindfulness interventions show promise, their effectiveness should be directly compared with that of other interventions such as CBT, exposure therapy, and other established interventions [80,81]. This would help determine the relative efficacy of MBIs in addressing PTSD, depression, and body image concerns in burn patients. This could also help in designing new integrated interventions. Moreover, the specific effects of compassion-based interventions should be explored, as the reviewed literature supports self-compassion as one of the main drivers of well-being in burn patients [49,54]. In general, understanding the effect of different mindfulness exercises on patients could help in tailoring more effective interventions targeting their specific needs, as multiple kinds of meditation exist [82], with specific neural and psychological effects [83,84]

Lastly, more studies are needed to evaluate whether mindfulness-based techniques influence biological markers of healing, including inflammation, collagen production, and immune function. For instance, research has shown that mindfulness practices can effectively reduce the cortisol levels, which is particularly relevant in burn recovery; as this mechanism supports stress regulation and promotes tissue healing [85]. Additionally, MBIs may modulate immune function and pain pathways, potentially contributing to improved wound healing and a reduction in the chronic pain frequently experienced by burn survivors [21,86]. This line of research could provide evidence for the direct physiological benefits of mindfulness. At the present moment, only scarce evidence exists that mindfulness and yoga exercises could help physical healing [86]. To advance our understanding of the effects of MBIs in this population, future research should specifically investigate these potential physiological mechanisms alongside psychosocial outcomes.

## 5. Conclusions

Mindfulness-based interventions represent a promising psychological tool for burn survivors, particularly to reduce distress, enhance pain management, and improve self-acceptance. However, the existing studies have methodological limitations, including small sample sizes, a lack of control groups, and limited follow-up periods. While mindfulness is a valuable complementary approach, future research should refine its application, explore its long-term benefits, and integrate it into comprehensive burn rehabilitation programs.

## Figures and Tables

**Table 1:** Quality assessment of the studies included in the systematic review based on the NIH study quality assessment tool.

	Sveen et al. (2017)	Conn et al. (2017)	Abd Elalem et al. (2018)	Ozdemir & Saritas (2019)	Hawkins et al. (2019)	Sheperd et al. (2019)	Nambi et al. (2021)	Al-Ghabeesh (2022)	Al-Ghabeesh & Mahmoud (2022)	Papamikrouli et al. (2023)	Al-Ghabeesh et al. (2024)	Sheperd et al. (2024)
Research question	1	1	1	1	1	1	1	1	1	1	1	1
Study population	1	1	1	1	1	1	1	1	1	1	1	1
Participation and drop-out	1	1	N	N	0	N	1	N	N	1	N	1
Participant selection	1	1	1	1	1	1	1	1	1	1	1	1
Sample size	0	1	1	1	1	0	1	0	0	0	1	1
Timeframe of the study	1	1	0	1	1	0	1	1	N	1	1	1
Reliable assessment and statistics	1	1	0	1	1	1	1	1	1	0	1	1
Follow-up assessment	0	0	0	0	0	0	1	0	0	1	0	1
Assessment of outcomes	1	1	1	1	1	1	1	1	1	1	1	1
Blindness	0	0	0	0	0	0	1	0	0	0	0	0
Confounding control	1	0	0	1	0	0	0	0	0	0	1	1
Quality rating (good, fair, or poor)	G	F	P	G	F	P	G	F	P	F	F	G

Note. Each column corresponds to a reviewed study, while each row corresponds to one of the key dimensions considered for the quality assessment. In addition, 1 indicates that the dimension was properly addressed or reported in the study, 0 indicates that the paper did not address properly a given dimension; N indicates that a dimension was not evaluable. The color code corresponds to the evaluation as follows: green = 1, red = 0, and yellow = not evaluable. The overall quality rating of each study was based on its total score as follows: poor (less than 6), fair (between 6 and 8), and good (higher than 8).

**Table 2:** Studies included in the systematic review (*n* = 14).

Year	Authors	Design	Sample	Intervention/ Instruments	Main Findings	Quality Evaluation
2017	Sveen et al.	IS (RCT)	62 parents of children with burn injuries. Mean age = 36.4; females = 42.	Six-week program including psychoeducational factors and mindfulness vs. waitlist control program.	The support program reduced symptoms of posttraumatic stress in parents at post-intervention and 3-month follow-up, but not at 12-month follow-up, with respect to the control group.	Good
2017	Conn et al.	IS with no control group	40 children with burn injuries. Mean age = 9.45 years; females = 7.	4-day yoga intervention (Burn Camp Yoga Kids program).	The program significantly reduced somatic and cognitive anxiety.	Fair
2018	Abd Elalem et al.	IS with no control group	34 adults in the burn unit. Mean age = 40.4; females = 18.	8-week self-care nursing intervention including mindfulness meditations, body scanning, and breathing exercises.	Improvement of self-esteem and QOL at post-intervention.	Poor
2019	Ozdemir & Saritas	IS (RCT)	110 burn patients. Mean age = 40.5 years; females = 58.	4-week program including 30 min of yoga 3 times a week vs. no practice (control).	Yoga Nidra practice significantly increased self-esteem and improved body image in burn patients with respect to the control group.	Good
2019	Hawkins et al.	CS	91 parents and primary caregivers of 71 children with burn injuries. Mean age = 33.62; females = 63.	Self-Compassion Scale—Short Form (SCS-SF)	Self-compassion was related to reduced depression and post-traumatic stress symptoms, but not anxiety.	Fair
2019	Sheperd et al.	CS	78 burns patients. Mean age = 45.2 years; females = 47.	Derriford Appearance Scale (DAS-24), Acceptance and Action Questionnaire II (AAQ-II), Cognitive Fusion Questionnaire (CFQ), Committed Action Questionnaire (CAQ-8), Five-Facet Mindfulness Questionnaire (FFMQ).	Increased appearance anxiety was related to reduced acceptance and cognitive defusion, as well as to reduced levels of various mindfulness facets.	Poor
2021	Nambi et al.	IS (RCT)	30 subjects with restrictive lung disease following circumferential burns of the chest. Mean age = 35.2 years; females = 8.	4-week program of yogic pranayama-based breathing exercise vs. 4-week diaphragmatic breathing exercise.	Pranayama breathing exercise showed more significant improvements in pain intensity, quality of life, pulmonary function, respiratory muscle activity, and exercise tolerance compared to conventional breathing exercise. The results were held at a three-month follow-up.	Good
2022	Al-Ghabeesh	CS	224 burn survivors. Mean age = 35.13 years; females = 98.	Mindful awareness and attention scale (MAAS), HADS.	Mindfulness was negatively associated with the level of psychological distress.	Fair
2022	Al-Ghabeesh & Mahmoud	CS	212 burn survivors. Mean age = 34.93 years; females = 93.	Mindful Awareness and Attention Scale (MAAS), Burn-Specific QOL (BSHS-B).	QOL was significantly and positively correlated with mindfulness, even while controlling for demographic and clinical variables.	Poor
2023	Papamikrouli et al.	IS with no control group	8 burn survivors (mean age = 49.4 years; females = 7) and 9 parents of children with burns (mean age = 52.2 years; female parents = 6).	8-week MBSR program	The intervention improved mindfulness skills and self-compassion in parents. Both groups reported increased personal goal scores immediately after the intervention and three months later.	Fair
2024	Al-Ghabeesh et al.	CS	212 burn survivors. Mean age = 34.93 years; females = 93	Kessler Psychological Distress Scale (K6), Multidimensional Scale of Perceived Social Support (MSPSS), Mindful Attention Awareness Scale (MAAS)	Mindfulness was related to reduced psychological distress while controlling for social support and demographic variables.	Fair
2024	Sheperd et al.	CS *	175 burns patients. Mean age = 42.2 years; females = 58.	Acceptance and Action Questionnaire (AAQ-II) and Self-Compassion Scale—Short Form (SCS-SF)	Acceptance and self-compassion at admission were associated with decreased appearance concerns cross-sectionally and prospectively at two- and six-month follow-up.	Good

Note. CS = cross-sectional study; IS = intervention study; QOL = quality of life; RCT = randomized controlled trial; MBSR = mindfulness-based stress reduction. * This study is listed as correlational but also includes a longitudinal assessment.

## Data Availability

No new data were created or analyzed in this study.
